# PTPRD silencing by DNA hypermethylation decreases insulin receptor signaling and leads to type 2 diabetes

**DOI:** 10.18632/oncotarget.4092

**Published:** 2015-05-25

**Authors:** Yng-Tay Chen, Wei-De Lin, Wen-Lin Liao, Ying-Ju Lin, Jan-Gowth Chang, Fuu-Jen Tsai

**Affiliations:** ^1^ Human Genetic Center, Department of Medical Research, China Medical University Hospital, China Medical University, Taichung, Taiwan; ^2^ School of Post Baccalaureate Chinese Medicine, China Medical University, Taichung, Taiwan; ^3^ Center for Personalized Medicine, China Medical University Hospital, Taichung, Taiwan; ^4^ Graduate Institute of Integrated Medicine, China Medical University, Taichung, Taiwan; ^5^ Graduate Institute of China Medical Science, China Medical University, Taichung, Taiwan; ^6^ Epigenome Research Center, China Medical University Hospital, Taichung, Taiwan; ^7^ Department of Laboratory Medicine, China Medical University Hospital, Taichung, Taiwan; ^8^ School of Medicine, China Medical University, Taichung, Taiwan; ^9^ Department of Medical Genetics, China Medical University Hospital, Taichung, Taiwan; ^10^ School of Chinese Medicine, China Medical University, Taichung, Taiwan; ^11^ Department of Health and Nutrition Biotechnology, Asia University, Taichung, Taiwan

**Keywords:** DNA methylation, DNMT1, PTPRD, type 2 diabetes

## Abstract

Genome-wide association study (GWAS) data showed that the protein tyrosine phosphatase receptor type delta (PTPRD) is associated with increased susceptibility to type 2 diabetes (T2D) in Han Chinese. A replication study indicated that PTPRD is involved in the insulin signaling pathway; however, the underlying mechanism remains unclear. We evaluated *PTPRD* expression in patients with T2D and controls. *PTPRD* expression levels were lower in patients and were correlated with the duration of the disease. Overexpression of the human insulin receptor PPARγ2 in HepG2 cells induced overexpression of *PTPRD* and the insulin receptor. *PTPRD* knockdown, using a shRNA, resulted in down-regulation of the insulin receptor. These results indicate that *PTPRD* activates PPARγ2 in the insulin signaling pathway. Similar results for *PTPRD* expression were found using a T2D mouse model. Silencing of *PTPRD* was caused by DNA methylation in T2D mice and patients, and correlated with *DNMT1* expression. Furthermore, we showed that a *DNMT1* SNP (rs78789647) was correlated with susceptibility to T2D. This study shows for the first time that DNMT1 caused PTPRD DNA hypermethylation and induced insulin signaling silencing in T2D patients. Our findings contribute to a better understanding of the crucial roles of these regulatory elements in human T2D.

## INTRODUCTION

Type 2 diabetes (T2D) is a complex disorder that is characterized by hyperglycemia, caused by impaired pancreatic β-cell function, decreased insulin action, and increased glucose output by the liver [1–3]. Both genetic and environmental factors contribute to the pathogenesis of T2D [4]. The disease is considered a polygenic disorder in which each genetic variant confers a partial and additive effect. Only 5–10% of the T2D cases are due to single-gene defects; these include maturity-onset diabetes of the young (MODY), insulin resistance syndromes, mitochondrial diabetes, and neonatal diabetes [5–7]. Inherited variations have been identified from studies of monogenic diabetes and have provided insights into β-cell physiology, insulin release, and action of insulin in fat, muscle, and liver [8].

Tsai *et al*. [9] initially identified the rs17584499 T allele in the protein tyrosine phosphatase receptor type delta (*PTPRD*) gene as the allele with the strongest association with type 2 diabetes in Han Chinese. Protein tyrosine phosphatases (PTPases) stimulate growth, proliferation, differentiation, cell cycle, and survival [10]. *PTPRD* is one of the most frequently inactivated genes across human cancers, including glioblastoma multiforme [11]. PTPRD was shown to be a tumor suppressor, as loss of PTPRD caused aberrant STAT3 activation in gliomas, resulting in glioma progression [12]. Epigenetic silencing of *PTPRD* was observed in glioblastoma [12]. The *PTPRD* genetic variant was suggested to be associated with progression to diabetes in Han Chinese, most likely through enhanced insulin resistance [13]. Recent studies have shown that these epigenetic factors contribute to the pathogenesis of T2D. However, until now, the exact roles of epigenetic factors and PTPRD in T2D onset and development remain unclear.

To gain insight into PTPRD function and the mechanism by which it activates the insulin signaling pathway, we examined the changes in *PTPRD* epigenetics in T2D.

## RESULTS

### Lower expression of *PTPRD* in patients with T2D

**Figure 1:**
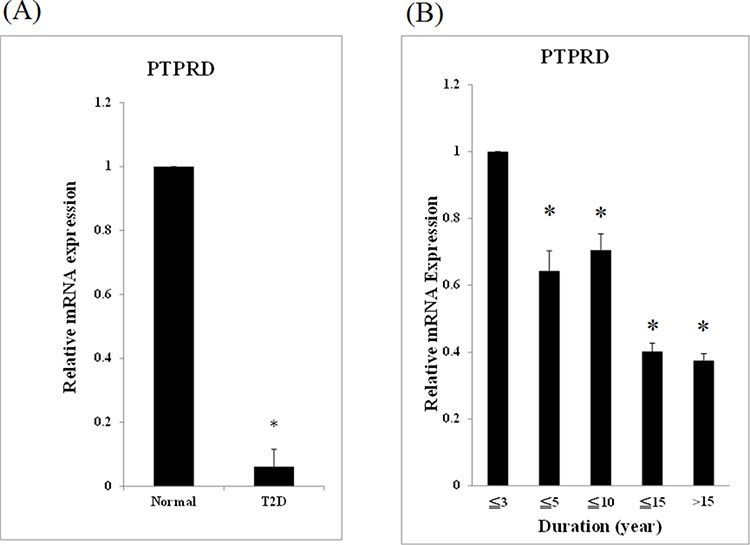
*PTPRD* mRNA was lower expression in T2D patients **A.** mRNA from 94 T2D patients and 98 normal controls was used for qRT-PCR. Expression levels are expressed relative to controls. **B.** The 94 patients with T2D were divided into 5 groups according to the disease duration: (1) less than 3 years (*N* = 19), (2) less than 5 years (*N* = 16), (3) less than 10 years (*N* = 27), (4) less than 15 years (*N* = 17), and (5) longer than 15 years (*N* = 15). The relative mRNA expression of group (2), (3), (4), and (5) versus to group (1) were 0.64 ± 0.06, 0.71 ± 0.05, 0.40 ± 0.03, and 0.37 ± 0.02, respectively (*P* < 0.05).

GWAS data previously indicated that PTPRD is involved in T2D. However, the exact role of PTPRD in T2D is still unclear. First, we checked *PTPRD* mRNA levels in blood samples of patients and controls. The relative *PTPRD* mRNA level was significantly lower in T2D patients than in controls (1 versus 0.07, *P* < 0.05, Fig. 1A). In addition, relative *PTPRD* mRNA expression levels were significantly lower in patients with T2D with disease duration of less than 5, 10, 15, and longer than 15 years than those of patients with disease duration less than 3 years. *PTPRD* mRNA levels decreased with the disease duration (1 versus 0.64, 0.71, 0.40, and 0.37 respectively, *P* < 0.05, Fig. 1B).

### PTPRD is involved in the insulin signaling pathway

**Figure 2:**
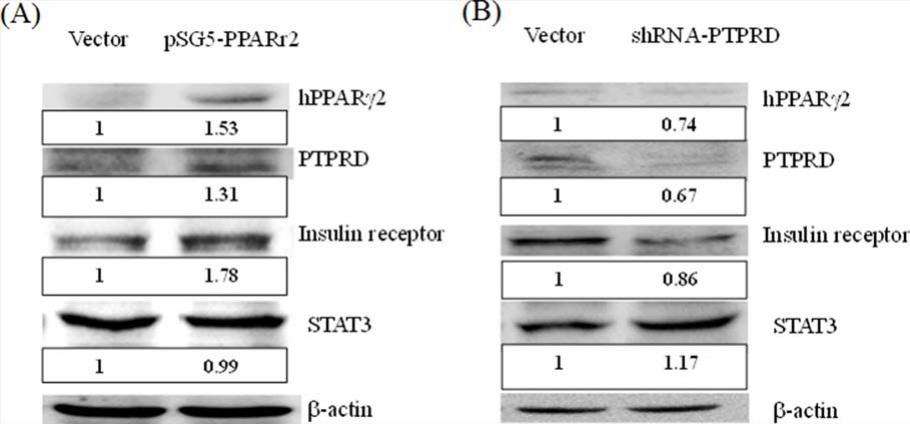
PTPRD is involved in the insulin signaling pathway **A.** HepG2 cells were transfected with pSG*5 vec*tor and with pSG5-h-PPARγ2 for 48 h. *PPAR*γ2 overexpression (1.53 fold versus vector only) induces the overexpression of *PTPRD* and insulin receptor 1.31 and 1.78 fold versus vector only, respectively. **B.** HepG2 cells were transfected with shRNA for 48 h. *PTPRD* knockdown (0.67 fold versus vector only) induced down-regulation of the *PPAR*γ2 and insulin receptor (0.74 and 0.86 fold versus vector only, respectively). Moreover, STAT3 was induced 1.17 fold versus vector only.

Little is known about the involvement of PTPRD in the insulin signaling pathway. Therefore, we used a cell model to analyze this further. We constructed a plasmid containing a fragment of human *PPAR*γ2 (pSG5-*hPPAR*γ2) for transient transfection of HepG2 cells. The results indicated that expression of human *PPAR*γ2 induced the overexpression of *PTPRD* and the insulin receptor (Fig. 2A). We used shRNA to knock down *PTPRD* expression in HepG2 cells and found that the insulin receptor was simultaneously down-regulated (Fig. 2B). These results indicate that PTPRD is involved in the insulin signaling pathway.

### DNA hypermethylation induces *PTPRD* silencing in T2D mice

**Figure 3:**
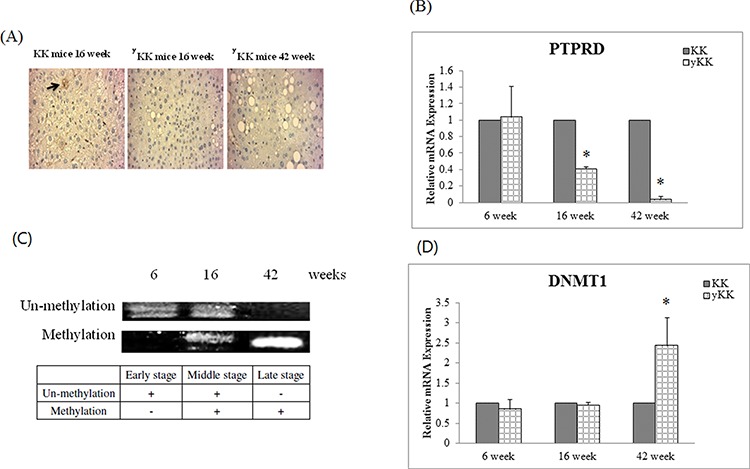
PTPRD protein was inhibition by T2D development duration in mice **A.** Immunocytochemical staining of liver tissues shows that PTPRD protein is expressed in 16-week-old KK mice, but not in 16- and 42-week-old ^y^KK mice. **B.** *PTPRD* mRNA expression in liver tissues is not different in 6-week-old KK and yKK mice. However, expression is significantly lower in 16- and 42-week-old ^y^KK mice than in KK mice. **C.** *PTPRD* DNA showed un-methylation in 6- and 16-week-old ^y^KK mice, but not in 42-week-old ^y^KK mice. *PTPRD* is methylated in liver tissues of 16- and 42-week-old ^y^KK mice. **D.** *DNMT1* expression by T2D development duration in mice. *DNMT1* expression is not different in 6- and 16-week-old KK and ^y^KK mice. In 42-week-old mice, *DNMT1* expression is significantly higher in ^y^KK than in KK mice (2.44 ± 0.7 fold, *P* < 0.05).

We used a T2D mouse model for a time course experiment. We used 6-week-old mice as representative for the early disease stage, 16-week-old for the middle stage, and 42-week-old for the late stage. Results showed that PTPRD protein expression was significantly lower in late stage mice compared with that of early stage, middle stage, and control mice (Fig. 3A). The relative *PTPRD* mRNA levels decreased with the disease stage (KK versus yKK at 6, 16, and 42 weeks were 1.04, 0.41, and 0.04 respectively, *P* < 0.05, Fig. 3B). These results indicated that the decrease in *PTPRD* expression was not caused by post-translational modification, but might be suppressed DNA transcription level. Therefore, we examined the methylation status of the *PTPRD* promoter, using methylation specific PCR (MSP). The result showed that the *PTPRD* promoter was methylated in middle and late stage T2D mice, but not in early stage T2D mice (Fig. 3C).

### DNMT1 induces *PTPRD* DNA methylation in diseased mice

Next, we wanted to analyze which DNA methyltransferase caused *PTPRD* methylation in T2D mice. Therefore, we checked *DNMT1*, *DNMT3A*, *DNMT3B*, and *DNMT3L* mRNA levels in diseased mice. Results indicated that *DNMT1* expression was significantly higher in later stage T2D mice (KK versus yKK at 6, 16, and 42 weeks were 0.86, 0.95, and 2.44 respectively, *P* < 0.05, Fig. 3D), but that of *DNMT3A*, *DNMT3B*, and *DNMT3L* was unaffected.

### *PTPRD* hypermethylation correlates with T2D progression

**Figure 4:**
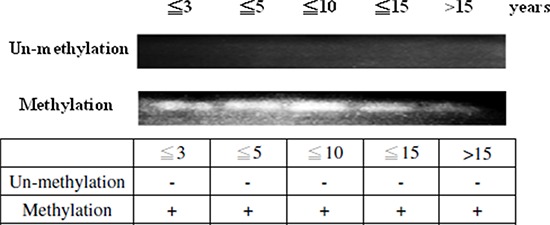
*PTPRD* methylation during disease development The 94 patients with T2D were divided into 5 groups according to the disease duration, (1) less than 3 years (*N* = 19), (2) less than 5 years (*N* = 16), (3) less than 10 years (*N* = 27), (4) less than 15 years (*N* = 17), and (5) longer than 15 years (*N* = 15). *PTPRD* is methylated in every disease stage in patients.

We then investigated *PTPRD* methylation during disease progression, using MSP. Patients with T2D with disease duration less than 3, 5, 10, 15 years, and longer than 15 years showed hypermethylation in the *PTPRD* DNA promoter site (Fig. 4). Hypermethylation was observed at the onset of the disease and throughout the disease progression.

### DNMT1 induces *PTPRD* DNA methylation in patients

**Figure 5:**
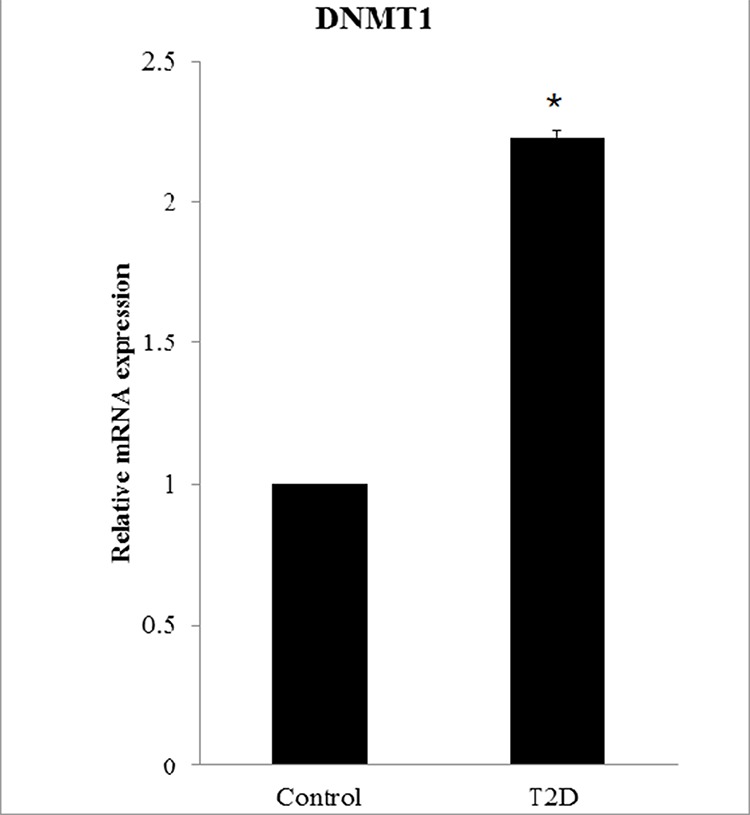
*DNMT1* overexpression in patients with T2D *DNMT1* expression is significantly higher in patients with T2D (*N* = 94) than in controls (*N* = 98). The relative mRNA expression level in patients is 2.23 ± 0.025.

In this study, the analysis of single nucleotide polymorphism (SNP) associated with DNA methyltransferases *DNMT1, DNMT3A, DNMT3B, and DNMT3L* was performed on 1924 patients with T2D and 3602 controls (Table S1–S4). For SNP rs78789647, the frequency of the “TT” genotype was higher in patients (0.52%) than in controls (0.28%) (Table 1). The frequency of the “TC” genotype was higher in patients (14.09%) than in controls (12.08%). In comparison with the “CC” genotype, the odds ratio (OR) of “TT” was 1.92 (95% CI = 0.80–4.63, *P* < 0.05). The allelic frequency of “T” was higher in patients (7.56%) than in controls (6.32%). In comparison to the “C” allele, the OR for the “T” allele was 1.21 (95% CI = 1.04–1.41, *P* < 0.05). All differences were found to be statistically significant. We found a *DNMT1* SNP (rs78789647) that correlated with susceptibility to T2D (Table 1). Moreover, *DNMT1* mRNA expression was higher in patients than in controls (relative expression was 2.23, *P* < 0.05, Fig. 5).

**Table 1:** Genotypic and allelic frequencies of DNMT1 genetic polymorphism in patients with T2D and controls

dbSNP ID		T2D	Control	OR (95% CI)	*P*-value
rs78789647	Genotype	Total = 1924 (%)	Total = 3602 (%)		
	TT	10(0.52)	10(0.28)	1.92(0.80–4.63)	0.035
	TC	271(14.09)	435(12.08)		
	CC	1643(85.39)	3157(87.64)	Ref	
	Allele frequency				
	T	291(7.56)	455(6.32)	1.21(1.04–1.41)	0.013
	C	3557(92.44)	6749(93.68)	Ref	

CI, confidence interval; OR, odds ratio.

^a^Genotype distribution between patients and control were calculated by 2 × 3 chi square test.

## DISCUSSION

GWAS data can reveal markers that may explain clinical and pathological specifics of T2D in a given population. Tsai *et al*. [9] found that the *PTPRD* gene was associated with susceptibility to T2D in Han Chinese. T2D risk alleles in *PPARγ2* (rs1801282) and *PTPRD* (rs17584499) are associated with the therapeutic efficacy of pioglitazone [14, 15]. In this study, we showed that *PTPRD* mRNA levels were lower in patients with T2D and that they were correlated with disease progression. Until now, no correlation of T2D disease with genetic and environmental factors or with epigenetic regulation has been found.

Cells overexpressing *PPARγ2* were used to examine PTPRD function in insulin signaling pathway in T2D. We found that *PPARγ2* expression caused *PTPRD* overexpression in an insulin receptor-sensitive manner. We also found that *PTPRD* knockdown induced the down-regulation of the insulin receptor. *PPARγ2* gene expression was previously shown to be associated with T2D [16]. PPARγ2 belongs to the nuclear hormone receptor superfamily of ligand-dependent transcription factors [17] and is mainly expressed in adipose tissue [18]. Thiazolidinedione (TZD) is an insulin-sensitizing drug, which is widely used for patients with T2D [19, 20] and works mainly as an agonist of the nuclear receptor PPARγ [21]. The binding of TZD to PPARγ2 activates the transcription of genes involved in glucose and lipid metabolism [22]. These results indicate that PTPRD is involved in the insulin signaling pathway. STAT3, a well-known oncoprotein [23], was inactivated by *PTPRD* activation and STAT3 was overexpression while PTPRD was inhibited [11], our results showed that PTPRD was well functional in the pathway.

KK-A^y^ mice, also known as Yellow KK obese mice, carry the lethal yellow obese (A^y^) mutation and develop diabetes of polygenic origin, show severe obesity, hypertriglyceridemia, hyperglycemia, hyperinsulinemia, and glucose intolerance by 8 weeks of age [24, 25]. Therefore, they are widely used as an experimental model for obesity and type 2 diabetes. In the current study, we used early stage mice, before 8 weeks of age, middle stage after 8 weeks of age, and later stage at 42 weeks of age for our experiments. We examined *PTPRD* expression at different time points during the disease progression. Results indicated that *PTPRD* mRNA and protein expression were lower in middle stage and later stage T2D mice than in controls. These results were similar to those obtained for patients with T2D. We found *PTPRD* promoter hypermethylation in middle and late disease stages in mice, but not in early stage and normal mice, similarly to the results found in patients. The *PTPRD* gene is frequently subject to cancer-specific epigenetic silencing via promoter CpG island DNA hypermethylation [12]. This suggests that *PTPRD* might be epigenetically silenced by DNA methylation in T2D as well. DNA methylation of CpG sites is the most common mechanism of epigenetic gene silencing. In cancer, aberrant methylation often results in the inactivation of tumor suppressor genes [26]. Several studies have identified tumor-specific methylation of CpG islands within the 5′ end of the regulatory region of the genes encoding the receptor-type PTPs such as *PTPRD* [12] in a wide range of adenocarcinomas, glioblastomas, and squamous cell carcinomas [27]. Our result is the first report to show the correlation between PTPRD and T2D disease development.

DNA methylation in humans occurs predominantly in the context of CpG dinucleotides [28]. The enzymes responsible for this modification, DNA methyltransferases (DNMTs), are well-characterized and conserved in mammals [28]. DNMTs fall under two categories: *de novo* and maintenance [29]. Patterns of DNA methylation are initially established by the *de novo* DNA methyltransferases, DNMT3A and DNMT3B, during the blastocyst stage of embryonic development [30, 31]. Our results showed that *DNMT3A*, *DNMT3B*, and *DNMT3L* expression was not different in T2D and control mice. However, *DNMT1* expression was significantly higher in T2D mice and patients than in controls. We also found a *DNMT1* SNP (rs78789647) that correlated with susceptibility to T2D. DNMT1 shows a preference for hemi-methylated DNA [32–34]. Both the establishment and maintenance of DNA methylation patterns are crucial for development, as DNMT3B- or DNMT1-deficiency in mice results in embryonic lethality [31, 35]. *De novo* DNA methylation in many human primary tumors is initiated within exon 1 of tumor-suppressor genes and subsequently spreads to the promoter region [36]. Using a multifaceted genomic analysis approach, we determined that *PTPRD* is inactivation via both genetic and epigenetic mechanisms in T2D.

In this study, we found the first evidence that PTPRD levels are significantly lower in patient with T2D, that this protein is involved in the insulin signaling pathway, and that it can be epigenetically silenced by DNA methylation. We also found that DNMT1 increases *PTPRD* DNA methylation during T2D development. These findings increase the understanding of the crucial roles of the regulatory elements in human T2D susceptibility.

## MATERIALS AND METHODS

### Patients and sample collection for quantitative real-time PCR (qRT-PCR) analysis

A total of 192 blood samples (98 controls and 94 patients with T2D) were collected. All individuals attended the China Medical University Hospital in Taichung and fulfilled the diagnostic criteria for T2D. Total RNA was isolated from human blood using the High Pure RNA Isolation Kit (Roche, Mannheim, Germany) according to the manufacturer's instructions. cDNA was synthesized from 1 μg total RNA, using the High Capacity cDNA reverse transcription kit (Applied Biosystems, Foster City, CA, USA), in a 20-μL reaction volume, according to the manufacturer's instructions. cDNA was diluted to 10 ng/L, and 1 μL cDNA was used for each qRT-PCR reaction in a final reaction volume of 10 μL. For quantification of gene expression with the ABI ViiA™ 7 Real-Time PCR System (Applied Biosystems), the FastStart Universal SYBR Green Master mix (Roche) was used. Primer sequences were as follows: *PTPRD* sense: 5′-TTTACACGAACACCCGTTGA-3′, antisense: 5′-CGGAGTCCGTAAGGGTTGTA-3′; and *GAPDH* sense: 5′-CAGCCTCAAGATCATC AGCA-3′, antisense: 5′-TGTGGTCATGAG TCCTTCCA-3′. This study was approved by the Human Studies Committee of China Medical University Hospital, and informed consent was obtained from either the participants or their parents.

### Genomic DNA extraction and MSP

Genomic DNA was extracted from peripheral blood leukocytes by using the Genomic DNA extraction kit (Qiagen, Valencia, CA, USA), according to the manufacturer's instructions. Bisulfite modification of genomic DNA was carried out using the EZ DNA methylation Kit (Zymo Research, Irvine, CA, USA). Primer sequences used for PTPRD MSP were as follows: 5′-TGTGGGGGTAGTGTTTTGTTTTG-3′; 5′-ACTCTC CCCACCAAAACTAACTAACA-3′; 5′-TGGGGGTAGC GTTTCGTTTC-3′; and 5′-CCCGCCGAAAC TAACTA ACG-3′ [10].

### Transfections

HepG2 cells were seeded at a density of 150, 000 cells per well on six-well culture plates and incubated until the culture reached 50–80% confluence. Cells were then transfected with either the empty pSG5 vector, pSG5-*hPPARγ2* (provided by Professor Haw-Wen Chen, Department of Nutrition, China Medical University, Taichung, Taiwan), or the lentiviral expression system for *PTPRD* shRNA (provided by the National RNAi Core Facility, Academia Sinica, Taiwan), by using the Xfect Transfection Reagent (Clontech, Palo Alto, CA, USA), according to the manufacturer's instructions. After 24 h of transfection, total RNA and protein were isolated from the cells.

### Protein extraction and western blots

The cells were homogenized in ice-cold radioimmunoprecipitation (RIPA) lysis buffer (Millipore, Temecula, CA, USA) with freshly added protease inhibitor and phosphatase inhibitor (FIVEphoton, San Diego, CA, USA). The homogenate was incubated on ice for 30 min and centrifuged at 13, 000 *g* for 30 min at 4°C. The supernatant was used for western blotting. Proteins (40 μg) were separated by SDS-PAGE using a 10% acrylamide resolving gel and transferred to a polyvinylidene difluoride membrane. Membranes were incubated in a blocking solution containing 5% nonfat dry milk and 0.1% Tween-20 in Tris-buffered saline, followed by incubation with a rabbit anti-PTPRD (1:1, 000; LifeSpan BioSciences, Seattle, WA, USA), anti-PPAR (1:1,000; LifeSpan BioSciences), anti-insulin receptor (1:1,000; GeneTex, Irvine, CA, USA), anti-STAT3 (1:1,000; GeneTex), and anti-actin (1:5, 000; GeneTex) polyclonal primary antibodies. Membranes were then incubated with horseradish peroxidase-conjugated goat anti-rabbit IgG (1:5,000; Jackson ImmunoResearch, West Grove, PA, USA) secondary antibody. Proteins were visualized using SuperSignal West Pico Chemiluminescent Substrate or SuperSignal West Femto Chemiluminescent Substrate (Thermo, Rockford, IL, USA).

### Animals

Four-week-old male KK and KK-Cg-*A^y^*/J mice were obtained from the Jackson Laboratory (Bar Harbor, Maine, USA). Six-, 16-, and 42-week-old mice were used for analyses. Animals were housed in individual cages and fed lab chow *ad libitum* (LabDiet 5k52, St. Louis, MO, USA). The animals were housed in a room with a conditioned temperature (22–25°C), relative humidity (50–70%), and photoperiod (12-h light/12-h dark). This study was approved by the Institutional Animal Care and Use Committee (IACUC) of China Medical University (IACUC: 102–217).

### Immunohistochemistry

Immunohistochemical staining of PTPRD was performed using rabbit anti-PTPRD (LifeSpan BioSciences) and a streptavidin-biotin-peroxidase complex. After deparaffinization and rehydration, formalin-fixed paraffin-embedded tissue sections were incubated in 3% H_2_O_2_ in distilled water for 30 min at room temperature, followed by antigen retrieval by boiling the sections in 0.01 M citrate buffer for 20 min. Sections were then washed in 50 mM Tris-HCl (pH 7.6) with 0.05% Tween for 2 min. To block non-specific binding, all sections were incubated with 5% nonfat dry milk for 30 min at room temperature. The slides were then incubated with the primary antibody anti-PTPRD (1:500) for 1 h at room temperature. The reaction was stopped by rinsing the sections with 0.01 M PBS. The sections were then incubated with biotinylated anti-mouse/rabbit IgG serum (secondary antibody), followed by treatment with a peroxidase-labeled streptavidin-biotin complex and diaminobenzidine substrate to visualize the positive cells. Finally, sections were counterstained with hematoxylin, prior to being mounted for examination by light microscopy. PTPRD-positive cells per area in the mouse liver were counted using the Q500MC Image Analysis System (Leica, Nussloch, Germany). PTPRD-positive cells were quantified in 20 fields from the cortex and 15 from the medulla at a magnification of 200 ×.

### Patients and sample collection for genotyping

We enrolled 1938 patients in this study. All individuals attended the China Medical University Hospital in Taichung and fulfilled the diagnostic criteria for T2D. To compare the prevalence of polymorphisms in patients with that in a healthy population, we downloaded the genotype frequency data of 3,602 healthy controls from the Taiwan Biobank (https://taiwanview.twbiobank.org.tw/taiwanview/dl.do). The SNPs in the target gene were queried from the NCBI dbSNP database (http://www.ncbi.nlm.nih.gov/snp). We obtained the SNPs of our gene of interest and compared the SNPs in the diseased and control group. Chi-square tests were used to calculate odds ratios and *P* values. The study was approved by the institutional review board of the China Medical University Hospital.

### Statistical analysis

Data were assembled using the Microsoft Excel software and analyzed using the SPSS 15.0 (SPSS, Chicago, IL, USA) or the GraphPad Prism version 3 (GraphPad Software, San Diego, CA, USA) statistical packages. All values were expressed as means (± standard deviation (SD)). Normality of the data was tested using the Kolmogorov-Smirnov test. Hierarchical gene analysis and heat maps were determined using a Pearson correlation matrix. Depending on the probability distribution pattern and the total number of subjects, parametric (normal distribution and ≥ 50 subjects) or non-parametric tests (skewed distribution or < 50 subjects) were used. Levels of significance were set at *P* < 0.05 (two-tailed).

## SUPPLEMENTARY TABLES



## References

[1] Stumvoll M, Goldstein BJ, van Haeften TW (2005). Type 2 diabetes: principles of pathogenesis and therapy. Lancet.

[2] Liao WL, Tsai FJ (2014). Personalized medicine in Type 2 Diabetes. BioMedicine.

[3] Gunasekaran U, Gannon M (2011). Type 2 diabetes and the aging pancreatic beta cell. Aging (Albany NY).

[4] Ozanne SE, Sandovivi I, Constancia M (2011). Maternal diet, aging and diabetes meet at a chromatin loop. Aging (Albany NY).

[5] Ledermann HM (1995). Maturity-onset diabetes of the young (MODY) at least ten times more common in Europe than previously assumed?. Diabetologia.

[6] Maassen JA, T Hart LM, Van Essen E, Heine RJ, Nijpels G, Jahangir Tafrechi RS, Raap AK, Janssen GM, Lemkes HH (2004). Mitochondrial diabetes: molecular mechanisms and clinical presentation. Diabetes.

[7] Fajans SS, Bell GI, Polonsky KS (2001). Molecular mechanisms and clinical pathophysiology of maturity-onset diabetes of the young. N Engl J Med.

[8] Moore AF, Florez JC (2008). Genetic susceptibility to type 2 diabetes and implications for antidiabetic therapy. Annu Rev Med.

[9] Tsai FJ, Yang CF, Chen CC, Chuang LM, Lu CH, Chang CT, Wang TY, Chen RH, Shiu CF, Liu YM, Chang CC, Chen P, Chen CH (2010). A genome-wide association study identifies susceptibility variants for type 2 diabetes in Han Chinese. PLoS Genet.

[10] Hunter T (2000). Signaling—2000 and beyond. Cell.

[11] Ortiz B, Fabius AW, Wu WH, Pedraza A, Brennan CW, Schultz N, Pitter KL, Bromberg JF, Huse JT, Holland EC, Chan TA (2014). Loss of the tyrosine phosphatase PTPRD leads to aberrant STAT3 activation and promotes gliomagenesis. Proc Natl Acad Sci U S A.

[12] Veeriah S, Brennan C, Meng S, Singh B, Fagin JA, Solit DB, Paty PB, Rohle D, Vivanco I, Chmielecki J, Pao W, Ladanyi M, Gerald WL (2009). The tyrosine phosphatase PTPRD is a tumor suppressor that is frequently inactivated and mutated in glioblastoma and other human cancers. Proc Natl Acad Sci U S A.

[13] Chang YC, Chiu YF, Liu PH, Shih KC, Lin MW, Sheu WH, Quertermous T, Curb JD, Hsiung CA, Lee WJ, Lee PC, Chen YT, Chuang LM (2012). Replication of genome-wide association signals of type 2 diabetes in Han Chinese in a prospective cohort. Clin Endocrinol.

[14] Pei Q, Huang Q, Yang GP, Zhao YC, Yin JY, Song M, Zheng Y, Mo ZH, Zhou HH, Liu ZQ (2013). PPAR-gamma2 and PTPRD gene polymorphisms influence type 2 diabetes patients' response to pioglitazone in China. Acta pharmacol Sin.

[15] Maruthur NM, Gribble MO, Bennett WL, Bolen S, Wilson LM, Balakrishnan P, Sahu A, Bass E, Kao WH, Clark JM (2014). The pharmacogenetics of type 2 diabetes: a systematic review. Diabetes Care.

[16] Altshuler D, Hirschhorn JN, Klannemark M, Lindgren CM, Vohl MC, Nemesh J, Lane CR, Schaffner SF, Bolk S, Brewer C, Tuomi T, Gaudet D, Hudson TJ (2000). The common PPARgamma Pro12Ala polymorphism is associated with decreased risk of type 2 diabetes. Nat Genet.

[17] Issemann I, Green S (1990). Activation of a member of the steroid hormone receptor superfamily by peroxisome proliferators. Nature.

[18] Auwerx J (1999). PPARgamma, the ultimate thrifty gene. Diabetologia.

[19] Willson TM, Lambert MH, Kliewer SA (2001). Peroxisome proliferator-activated receptor gamma and metabolic disease. Annu Rev Biochem.

[20] Zimmet P (2002). Addressing the insulin resistance syndrome: a role for the thiazolidinediones. Trends Cardiovasc Med.

[21] Soccio RE, Chen ER, Lazar MA (2014). Thiazolidinediones and the Promise of Insulin Sensitization in Type 2 Diabetes. Cell Metab.

[22] Miyazaki Y, Mahankali A, Matsuda M, Mahankali S, Hardies J, Cusi K, Mandarino LJ, DeFronzo RA (2002). Effect of pioglitazone on abdominal fat distribution and insulin sensitivity in type 2 diabetic patients. J Clin Endocrinol Metab.

[23] Bromberg JF, Wrzeszczynska MH, Devgan G, Zhao Y, Pestell RG, Albanese C, Darnell JE (1999). Stat3 as an oncogene. Cell.

[24] Iwatsuka H, Shino A, Suzuoki Z (1970). General survey of diabetic features of yellow KK mice. Endocrinol Jpn.

[25] Castle CK, Colca JR, Melchior GW (1993). Lipoprotein profile characterization of the KKA(y) mouse, a rodent model of type II diabetes, before and after treatment with the insulin-sensitizing agent pioglitazone. Arterioscler Thromb.

[26] Esteller M (2008). Epigenetics in cancer. N Engl J Med.

[27] Jacob ST, Motiwala T (2005). Epigenetic regulation of protein tyrosine phosphatases: potential molecular targets for cancer therapy. Cancer Gene Ther.

[28] Law JA, Jacobsen SE (2010). Establishing, maintaining and modifying DNA methylation patterns in plants and animals. Nat Rev Genet.

[29] Goll MG, Bestor TH (2005). Eukaryotic cytosine methyltransferases. Annu Rev Biochem.

[30] Okano M, Xie S, Li E (1998). Cloning and characterization of a family of novel mammalian DNA (cytosine-5) methyltransferases. Nat Genet.

[31] Okano M, Bell DW, Haber DA, Li E (1999). DNA methyltransferases Dnmt3a and Dnmt3b are essential for de novo methylation and mammalian development. Cell.

[32] Bestor TH, Ingram VM (1983). Two DNA methyltransferases from murine erythroleukemia cells: purification, sequence specificity, and mode of interaction with DNA. Proc Natl Acad Sci U S A.

[33] Bestor T, Laudano A, Mattaliano R, Ingram V (1988). Cloning and sequencing of a cDNA encoding DNA methyltransferase of mouse cells. The carboxyl-terminal domain of the mammalian enzymes is related to bacterial restriction methyltransferases. J Mol Biol.

[34] Hermann A, Goyal R, Jeltsch A (2004). The Dnmt1 DNA-(cytosine-C5)-methyltransferase methylates DNA processively with high preference for hemimethylated target sites. J Biol Chem.

[35] Li E, Bestor TH, Jaenisch R (1992). Targeted mutation of the DNA methyltransferase gene results in embryonic lethality. Cell.

[36] Nguyen C, Liang G, Nguyen TT, Tsao-Wei D, Groshen S, Lubbert M, Zhou JH, Benedict WF, Jones PA (2001). Susceptibility of nonpromoter CpG islands to de novo methylation in normal and neoplastic cells. J Natl Cancer Inst.

