# Supplementary material for: PTPRD silencing by DNA hypermethylation decreases insulin receptor signaling and leads to type 2 diabetes
Source: Oncotarget. 2015 May 25;6(15):12997–3005. doi: 10.18632/oncotarget.4092 (PMC4536994; doi:10.18632/oncotarget.4092)
Supplement: Supplementary file 1 [file oncotarget-06-12997-s001.pdf]

SUPPLEMENTARY TABLES

Supplementary Table S1. Genotypic and allelic frequencies of DNMT1 genetic polymorphisms in T2D patients and controls

		Type 2 diabetes	Healthy control	*P*	OR (95% CI)
**Genotype frequency**		**N (%)**	**N (%)**		
rs78789647	TT	10 (0.52)	10 (0.28)		1.921 (0.798–4.626)
	TC	271 (14.09)	435 (12.08)	0.035*	1.197 (1.017–1.409)
	CC	1643 (85.4)	3157 (87.65)		1
s16999593	CC	106 (5.51)	171 (4.8)		1.110 (0.862–1.428)
	CT	610 (31.7)	1226 (34.4)	0.088	0.891 (0.790–1.004)
	TT	1208 (62.79)	2163 (60.76)		1
rs2114724	TT	144 (7.48)	306 (8.5)		0.833 (0.674–1.031)
	TC	754 (39.17)	1477 (41.00)	0.100	0.904 (0.805–1.016)
	CC	1027 (53.35)	1819 (50.50)		1
rs7253062	AA	122 (6.34)	198 (5.50)		1.181 (0.932–1.497)
	AG	699 (36.35)	1290 (35.82)	0.359	1.039 (0.924–1.168)
	GG	1102 (57.31)	2113 (58.68)		1
**Allele frequency**					
rs78789647	T	291 (7.56)	455 (6.32)	0.013*	1.213 (1.042–1.414)
	C	3557 (92.44)	6749 (93.68)		1
rs16999593	C	822 (21.36)	1568 (22.02)	0.424	0.962 (0.874–1.058)
	T	3026 (78.64)	5552 (77.98)		1
rs2114724	T	1042 (27.06)	2089 (29.00)	0.032	0.909 (0.833–0.992)
	C	2808 (72.94)	5115 (71.00)		1
rs7253062	A	943 (24.52)	1686 (23.41)	0.192	1.063 (0.970–1.165)
	G	2903 (75.48)	5516 (76.59)		1

CI, confidence interval; OR, odds ratio.

[a]Genotype distribution between patients and control were calculated by 2 × 3 chisquare test.

Supplementary Table S2. Genotypic and allelic frequencies of DNMT3A genetic polymorphisms in T2D patients and controls

		Type 2 diabetes	Healthy control	*P*	OR (95% CI)
**Genotype frequency**		**N (%)**	**N (%)**		
rs11887120	CC	460 (23.93)	889 (24.70)		0.890 (0.762–1.041)
	CT	943 (49.06)	1817 (50.49)	0.206	0.893 (0.781–1.021)
	TT	519 (27.00)	893 (24.81)		1
rs11892646	TT	163 (8.49)	336 (9.33)		0.894 (0.730–1.095)
	TC	788 (41.06)	1481 (41.13)	0.556	0.981 (0.873–1.102)
	CC	968 (50.44)	1784 (49.54)		1
rs12987103	TT	104 (5.40)	184 (5.11)		1.092 (0.850–1.404)
	TC	695 (36.10)	1238 (34.41)	0.358	1.085 (0.965–1.220)
	CC	1126 (58.49)	2176 (60.48)		1
rs12991495	CC	22 (1.14)	41 (1.14)		0.997 (0.592–1.679)
	CT	318 (16.54)	620 (17.21)	0.816	0.953 (0.822–1.105)
	TT	1583 (82.32)	2941 (81.65)		1
rs13428812	GG	109 (5.66)	204 (5.66)		1.026 (0.804–1.309)
	GA	728 (37.82)	1309 (36.34)	0.544	1.068 (0.950–1.200)
	AA	1088 (56.52)	2089 (58.00)		1
rs1465825	CC	289 (15.02)	562 (15.61)		0.889 (0.752–1.052)
	CT	888 (46.15)	1746 (48.50)	0.097	0.880 (0.779–0.993)
	TT	747 (38.83)	1292 (35.89)		1
rs6711622	AA	82 (4.28)	136 (3.78)		1.166 (0.878–1.546)
	AG	640 (33.37)	1153 (32.02)	0.339	1.073 (0.952–1.209)
	GG	1196 (62.36)	2312 (64.20)		1
rs6722613	AA	219 (11.38)	400 (11.11)		1.032 (0.858–1.240)
	AG	831 (43.17)	1552 (43.10)	0.945	1.009 (0.897–1.135)
	GG	875 (45.45)	1649 (45.79)		1
rs7575625	GG	99 (5.16)	183 (5.08)		1.056 (0.818–1.362)
	GA	700 (36.50)	1234 (34.27)	0.234	1.107 (0.984–1.245)
	AA	1119 (58.34)	2184 (60.65)		1
rs7586294	CC	263 (13.68)	473 (13.14)		1.080 (0.907–1.285)
	CT	902 (46.91)	1655 (45.97)	0.554	1.058 (0.939–1.193)
	TT	758 (39.42)	1472 (40.89)		1
rs7587636	AA	196 (10.22)	353 (9.80)	0.673	1.069 (0.882–1.295)
	AG	810 (42.23)	1492 (41.43)		1.045 (0.930–1.175)

(*Continued*)

		Type 2 diabetes	Healthy control	*P*	OR (95% CI)
	GG	912 (47.55)	1756 (48.76)		1
rs7594432	TT	434 (22.59)	857 (23.79)		0.872 (0.746–1.019)
	TC	934 (48.62)	1793 (49.78)	0.160	0.897 (0.786–1.023)
	CC	553 (28.79)	952 (26.43)		1
rs77993651	AA	93 (4.83)	181 (5.03)		0.943 (0.727–1.224)
	AG	666 (34.62)	1278 (35.52)	0.725	0.957 (0.851–1.076)
	GG	1165 (60.55)	2139 (59.45)		1
**Allele frequency**		**N (%)**	**N (%)**		
rs11887120	C	1863 (48.47)	3595 (49.94)	0.139	0.943 (0.872–1.019)
	T	1981 (51.53)	3603 (50.06)		1
rs11892646	T	1114 (29.03)	2153 (29.89)	0.341	0.959 (0.880–1.045)
	C	2724 (70.98)	5049 (70.11)		1
rs12987103	T	903 (30.64)	1606 (28.73)	0.174	1.067 (0.972–1.170)
	C	2947 (76.55)	5590 (77.68)		1
rs12991495	C	362 (9.41)	702 (9.74)	0.573	0.962 (0.842–1.100)
	T	3484 (90.59)	6502 (90.26)		1
rs13428812	G	946 (24.57)	1717 (23.83)	0.388	1.041 (0.950–1.140)
	A	2904 (75.43)	5487 (76.17)		1
rs1465825	C	1466 (38.10)	2870 (39.86)	0.071	0.929 (0.857–1.006)
	T	2382 (61.90)	4330 (60.14)		1
rs6711622	A	804 (20.96)	1425 (19.79)	0.144	1.075 (0.976–1.184)
	G	3032 (79.04)	5777 (80.21)		1
rs6722613	A	1269 (32.96)	2352 (32.66)	0.746	1.014 (0.933–1.102)
	G	2581 (67.04)	4850 (67.34)		1
rs7575625	G	898 (23.41)	1600 (22.22)	0.154	1.070 (0.975–1.175)
	A	2938 (76.59)	5602 (77.78)		1
rs7586294	C	1428 (37.13)	2601 (36.13)	0.296	1.044 (0.963–1.133)
	T	2418 (62.87)	4599 (63.87)		1
rs7587636	A	1202 (31.33)	2198 (30.52)	0.377	1.039 (0.955–1.131)
	G	2634 (68.67)	5004 (69.48)		1
rs7594432	T	1802 (46.90)	3507 (48.68)	0.075	0.931 (0.861–1.007)
	C	2040 (53.10)	3697 (51.32)		1
rs77993651	A	852 (22.14)	1640 (22.79)	0.437	0.963 (0.877–1.058)
	G	2996 (77.86)	5556 (77.21)		1

CI, confidence interval; OR, odds ratio.
[a]Genotype distribution between patients and control were calculated by 2 × 3 chisquare test.

Supplementary Table S3. Genotypic and allelic frequencies of DNMT3B genetic polymorphisms in T2D patients and controls

		Type 2 diabetes	Healthy control	*P*	OR (95% CI)
**Genotype frequency**		**N (%)**	**N (%)**		
rs4911262	GG	7 (0.36)	15 (0.42)		0.876 (0.356–2.152)
	GA	241 (12.53)	444 (12.33)	0.936	1.019 (0.861–1.204)
	AA	1675 (87.10)	3143 (87.26)		1
rs6058894	TT	7 (0.36)	12 (0.33)		1.092 (0.429–2.779)
	TC	239 (12.45)	450 (12.51)	0.981	0.994 (0.841–1.176)
	CC	1674 (87.19)	3134 (87.15)		1
rs6119954	AA	195 (10.16)	368 (10.22)		0.977 (0.806–1.183)
	AG	834 (43.44)	1591 (44.18)	0.843	0.966 (0.859–1.086)
	GG	891 (46.41)	1642 (45.60)		1
rs6141813	GG	282 (14.66)	509 (14.14)		1.022 (0.862–1.211)
	GA	865 (44.98)	1659 (46.10)	0.709	0.961 (0.853–1.084)
	AA	776 (40.35)	1431 (39.76)		1
**Allele frequency**					
rs4911262	G	255 (6.63)	474 (6.58)	0.919	1.008 (0.861–1.180)
	A	3591 (93.37)	6730 (93.52)		1
rs6058894	T	253 (6.59)	474 (6.59)	0.997	0.9997 (0.854–1.171)
	C	3587 (93.41)	6718 (93.41)		1
rs6119954	A	1224 (31.87)	2327 (32.31)	0.641	0.980 (0.901–1.066)
	G	2616 (68.13)	4875 (67.69)		1
rs6141813	G	1429 (37.16)	2677 (37.19)	0.971	0.998 (0.921–1.083)
	A	2417 (62.84)	4521 (62.81)		1

CI, confidence interval; OR, odds ratio.
[a]Genotype distribution between patients and control were calculated by 2 × 3 chisquare test.

Supplementary Table S4. Genotypic and allelic frequencies of DNMT3L genetic polymorphism in T2D patients and controls

		Type 2 diabetes	Healthy control	*P*	OR (95% CI)
**Genotype frequency**		**N (%)**	**N (%)**		
rs2838537	GG	73 (3.80)	121 (3.36)		1.168 (0.867–1.574)
	GT	630 (32.78)	1121 (31.12)	0.270	1.088 (0.965–1.226)
	TT	1219 (63.42)	2360 (65.52)		1
**Allele frequency**					
rs2838537	G	776 (20.19)	1363 (18.92)	0.108	1.084 (0.982–1.196)
	T	3068 (79.81)	5841 (81.08)		1

CI, confidence interval; OR, odds ratio.

[a]Genotype distribution between patients and control were calculated by 2 × 3 chisquare test.
